# Functional and cognitive correlates of typing speed in a large U.S. panel study

**DOI:** 10.1038/s41598-026-36500-7

**Published:** 2026-01-21

**Authors:** Raymond Hernandez, Stefan Schneider, Margaret Gatz, Deborah Finkel, Sean Minns, Bart Orriens, Ying Liu, Arie Kapteyn

**Affiliations:** 1 Dornsife Center for Economic & Social Research, University of Southern California, Los Angeles, CA USA; 2 Department of Psychology, University of Southern California, Los Angeles, CA USA; 3 Leonard Davis School of Gerontology, University of Southern California, Los Angeles, CA USA; 4 Institute for Gerontology, Jönköping University, Jönköping, Sweden

**Keywords:** Public health, Epidemiology, Population screening, Human behaviour

## Abstract

Typing has become increasingly integral to everyday functioning. To comprehensively understand people’s level of functioning, researchers may also want to consider typing performance in addition to performance of other classical activities of daily living like dressing, shopping, or managing a budget. Classical typing tests are too burdensome to administer in panel studies. We examined functional and cognitive correlates of a one sentence typing speed test to examine if this brief measure could provide meaningful information. A sample of 10,613 adult participants in the Understanding America Study (UAS), a nationally representative longitudinal online survey panel, completed surveys, cognitive tests, and the typing test. Intraclass correlation coefficients of 0.79 for computer typing speed and 0.63 for smartphone typing speed suggested high test–retest stability for the brief typing test across 2 years. Faster typing speed was generally associated with better cognitive functioning across domains, fewer reported difficulties with basic and instrumental activities of daily living, and a lower likelihood of several self-reported illnesses. After adjustment for demographics, the correlation magnitudes were attenuated but still generally in the expected directions. Typing speed as captured by a single sentence typing speed test appeared to be sensitive to several measures relevant to everyday functioning.

**Supplementary Information:**

The online version contains supplementary material available at 10.1038/s41598-026-36500-7.

## Introduction

Typing on devices like computers, smartphones, and tablets has become a pervasive mode of communication worldwide^1,2^. Aside from paid employment, typing has become integral for a variety of other activities including accessing personal medical records^3^, online shopping^4^, and socializing^5^. Thus, adding typing performance to the repertoire of commonly assessed activities of daily living (e.g., showering and mobility within the home, managing a budget) may enrich understanding of everyday functioning difficulties. Typing speed often serves as a metric of typing performance and traditional typing tests involve copying text as quickly and accurately as possible^2^. Inferring performance from speed has often been of interest, such as assessment of functional mobility with gait speed^6^.

Typing speed may also have utility as a digital biomarker^7^. In related research, phone keystrokes and swipes have been used to infer cognitive ability^8^, phone accelerometer and call activity to assess social anxiety severity^9^, and mouse movements patterns as a potential marker of mild cognitive impairment^10^. Typing has perceptual, cognitive, and motor requirements so typing speed may be interpreted as reflective of some combination of these skills^11^. However, typing speed may serve as a metric of daily functioning in itself, especially in the modern world. If typing is too slow, people would have difficulties with a variety of now everyday activities including texting, browsing the Internet, and social media use.

Assessing typing performance via typing speed in large online survey panels could allow investigation of several questions of relevance to epidemiology and public health surveillance generally. For instance, it could allow investigation of which chronic conditions are associated with lower typing performance, which could advance understanding of the type of support different diagnostic groups require when trying to access resources online such as those relevant to governmental aid and health insurance. As one more example, having data on typing speed could also allow examination of demographic differences in typing performance. Any disparities could potentially be interpreted as a difference in human capital (i.e., skills, knowledge, and experience possessed by individuals) with possible well-being implications.

Additionally, a measure of typing speed could serve as an important control variable for online cognitive tests with a typing component. For example, the online version of the Picture Vocabulary test, which assesses word knowledge^12^, involves typing the correct name of a picture. Metrics of typing speed would allow for examining associations between word knowledge and other variables, after controlling for potentially confounding effects of typing speed.

In this paper, we examine the functional correlates of a one sentence typing speed test. A typing speed test that is very brief (i.e., copying only one sentence) may be most feasible to administer in online panel studies. Traditional typing speed tests can require between one to five minutes to complete, but this can be too long given the multitude of surveys participants must often complete in panel studies. Thus, we examine whether an extremely brief typing speed test can provide a meaningful performance assessment despite its brevity.

We hypothesized that faster typing speed on the brief typing test should be associated with better cognitive functioning, greater ability to engage in activities of daily living, and a lower probability of reporting any diagnoses of chronic illnesses. Typing involves an interplay of cognitive and motor skills^2,11^ with steps including the perception of meaningful chunks of information (e.g., text to be typed) and executing the movement necessary to press the correct keys^2,11^. Consistent with this, associations have been found between poorer typing speed and a greater likelihood of cognitive impairment^13^, fine motor impairment (which is highly related to activities of daily living like dressing and writing)^13,14^, and the likelihood of diagnoses such as Parkinson’s disease^15^. Many diagnoses have associations with cognitive/visual and fine motor skills, and thus may impact typing speed. For instance, impaired visual perceptual skills have been associated with hypertension^16^ and diabetes^17^ which could lead to slower typing speed.

We consider typing speed on a computer and typing speed on a smartphone separately, as they may not be entirely comparable tasks. For instance, typing on a smartphone typically occurs on small screens, so it has typing styles distinct from those seen in computer keyboarding, with two common approaches being one or two thumb typing^18,19^. On computers it is more feasible to engage in typing using all fingers (i.e., touch typing) given the use of a large dedicated keyboard.

## Methods

Data analyzed for this paper were collected from the Understanding America Study (UAS), a nationally representative longitudinal online survey panel of U.S. adults aged 18 and older all of whom provide informed consent prior to participation^20,21^. Participants in the UAS were recruited with address-based sampling. The randomly selected participants were sent a recruitment letter and asked to follow through with the enrollment instructions if interested in taking part^21^. To ensure adequate sampling of typically underrepresented racial groups and residents of rural areas, these groups were oversampled. Test–retest stability analyses were based on data from a subset of participants who completed the typing test twice (with an average of 2 years between tests) and utilized the same device in each trial. The study was approved by the Biomedical Research Alliance of New York Institutional Review Board (IRB). All methods were performed in accordance with the relevant guidelines and regulations of the IRB.

### Measures

#### Typing speed test

The typing speed test was adapted from the Health and Retirement Study^22^ Internet Survey (a sub-study of the HRS). Participants were presented with the following instructions: “Next, we would like you to type the sentence below as quickly as you can. When you are ready, please begin typing in the box, then select ‘Next’ when you are finished.” For participants who preferred English, the sentence presented was the following pangram, a sentence that contains all the letters of the alphabet: “The quick brown fox jumps over the lazy dog.” Participants who preferred Spanish were presented with the Spanish pangram “Cada vez que trabajo, Felix me paga un whisky.” Participants were allowed to complete the whole survey containing the typing speed test on a device of their choice: computer, smartphone, or tablet. Only typing tests that were completed on computers or smartphones (about 97% of observations) were considered in analyses, and 55% of this sample used a computer.

*Adjusted typing speed*, or adjusted words per minute (AWPM), was computed as the product between typing speed and accuracy^2^. This metric was used as the primary measure of typing speed because it considered both speed and accuracy of keyboarding. In analyses it was log-transformed to correct for positive skew in the distribution. From here on, our use of AWPM or the phrase “typing speed” refers to adjusted typing speed. Refer to Supplementary Text S1 for additional information on the typing test.

#### Functioning relevant measures

Several functioning relevant measures were examined to assess associations between typing speed and functioning.

*Cognitive measures* A variety of cognitive tests are administered in the UAS. The Figure Identification task (perceptual speed)^23^, Stop and Go Switch Test (choice reaction time, response inhibition, task switching)^24^, Serial 7s (attention and working memory)^25^, Number Series (fluid intelligence)^12^, Verbal Analogies (fluid intelligence)^12^, Picture Vocabulary (word knowledge)^12^, Immediate and Delayed Word Recall (episodic memory)^25^, and Box Clicking Test (visual motor integration)^26^ were all utilized in analyses. Please refer to Supplementary Text S1 for more detailed descriptions of the cognitive tests.

Additionally, composite scores for general cognition, perceptual speed, and memory were computed. Aligned with results from exploratory factor analysis, the general intelligence score used results from the Number Series, Verbal Analogies, and Picture Vocabulary tests. The speed score used results from the Figure Identification and Stop and Go tasks. Finally, the memory score was from the immediate and delayed word recall tasks. Please refer to Supplementary Text S1 and Supplementary Table S1 for additional details.

*Activities of daily living* Difficulties with basic activities of daily living (BADLs) and/or instrumental activities of daily living (IADLs) were assessed with questions adopted from the HRS: “Because of a health or memory problem do you have any difficulty with (BADL/IADL)? Exclude any difficulties that you expect to last less than three months.” The response options were “yes”, “no”, or “can’t do”. If a participant responded with “yes” or “can’t do”, then this was coded as a 1 indicating difficulty, while 0 was indicative of no difficulty. BADLs inquired about were dressing, eating, walking, getting in/out of bed, using the toilet, and bathing. IADLs asked about were meal preparation, making a phone call, shopping for groceries, taking medications, house/yard work, and money management.

*Self-reported diagnoses* Participants were asked “Has a doctor ever told you that you have (diagnosis)?” which was used in the Health and Retirement Study. Conditions covered were diabetes, arthritis, stroke, dementia, heart condition, hypertension, cancer, lung disease, and emotional/psychiatric problem.

*Other* Quality of eyesight was assessed with the item “Is your eyesight excellent, very good, good, fair, or poor—using glasses or corrective lens as needed?” Self-rated computer/smartphone skills was measured with the item “How would you rate your (device type) skill level?”.

### Statistical analyses

Demographic characteristics were computed separately for participants using a computer and those using a smartphone. To visually examine speed differences by age, two-year age bins from age 18 to 81 were first created (e.g., 18 to 19, 20 to 21, etc.). The size of the bins and age range were chosen to ensure that at least 50 individuals were represented in each age bin. Next, for each age bin, the average AWPM and the corresponding 95% confidence interval were computed, separately for computer and smartphone users. The averages and their confidence intervals were then plotted on a graph of age versus AWPM, by device.

To examine magnitude of cohort relative to aging effects on typing speed, for the subsample of participants that completed the typing speed test twice on the same device, a multilevel model was specified where typing speed was regressed on age at both the within and between person levels (separately for the computer and phone samples). To deconflate the within and between person effects of age on typing speed, at the within-person level age was person mean centered while at the between person level the person-mean average of age was used as a predictor^27^. Multilevel regression estimates were examined instead of multilevel correlations, as the former are more comparable across levels. The magnitude of the unstandardized between person estimate for age relative to its within-person estimate provides insight on the magnitude of cohort relative to aging effects on typing speed.

#### Stability

Test–retest stability of AWPM was computed for a subset of participants who completed the typing speed test twice on the same device, separately for computers (n = 1909) and smartphones (n = 1636). The intraclass coefficient (ICC) was computed to assess stability, which can be interpreted with the following guidelines: poor (0 to 0.19), fair (0.20 to 0.39), moderate (0.40 to 0.59), substantial (0.60 to 0.79), and almost perfect (0.80 to 1.0)^28^. The average amount of time between tests was 2.0 years, with a range from 1.0 year to 3.1 years. Thus, true change in typing speed (e.g., from aging) could decrease stability relative to a situation where the tests were administered a few days or weeks apart. This stability measure may also be interpreted as lower bound of test–retest reliability. That is, if the typing speed tests were administered closer together (e.g., a few days apart) like a traditional assessment of test–retest reliability, then one would expect the stability to be higher compared to 1 to 3 years separating tests.

#### Correlations with functioning relevant measures

To examine if typing speed showed the expected associations with other measures, correlations were computed, separately for people who completed the typing speed test on a computer and on a smartphone. The distributions of all continuous variables were checked for normality, after which all four measures of the Stop & Go test were log-transformed to normalize their distributions. Further, the majority of participants (66%) had a full score on the Serial 7s task, so it was converted to a binary variable indicating if participants had a fully correct score on it or not. The following guidelines were used to interpret the magnitudes of correlations: 0.1 small, 0.3 medium, and 0.5 large^29^. Pearson correlations were computed to examine associations between typing speed and continuous variables (e.g., perceptual speed), while biserial correlations were computed to examine associations between typing speed and binary variables (e.g., having a diagnosis of hypertension or not). Partial correlations were also computed where typing speed and all other variables were adjusted by the following demographic characteristics: age, gender, race, ethnicity, working status, education, income, and preferred language. In sensitivity analyses, a Benjamin Hochberg *p*-value adjustment^30^ was applied to account for the multiple comparisons, separately by device type for the demographic adjusted and unadjusted correlations.

In exploratory analyses we examined correlations between typing speed and functioning measures for only adults aged 65 and older as functional impairments and various diagnoses are typically more common in older adults relative to younger ones^31^.

All analyses were conducted with M*plus* version 8.11^32^ through the statistical software R^33^ and the R package MplusAutomation^34^. Missing data were addressed by using a robust maximum likelihood estimator in M*plus* models, which assumes that data is missing at random.

## Results

Analyses were conducted on 10,613 participants, whose demographic characteristics are summarized in Table 1. The typing speed test was completed on a computer by 55% of participants included in analyses, and on the smartphone by 45%. Computer and smartphone users showed statistically significant differences on all participant characteristics examined. For instance, the smartphone group was significantly younger, comprised more females, lower SES, and a greater proportion of individuals identifying as Hispanic or Latino. In terms of functional and diagnostic characteristics, the smartphone group reported BADL/IADL issues and various diagnoses with slightly lower frequency as compared to the computer group (Table 2). In both groups, participants aged 65 and older reported BADL/IADL issues and diagnoses with greater frequency compared to the whole sample. The subgroup of participants that completed the typing speed twice on the same device (n = 3545) had demographic characteristics very similar to the whole sample (Supplementary Table S2). For the computer and smartphone samples of this test–retest group, the proportions for the levels of all the categorical demographic variables were near identical to the proportions observed for the full computer and smartphone samples respectively.

**Table 1:** Demographic characteristics for computer (n = 5835) and smartphone (n = 4778) samples. To test if the samples differed by continuous variables like age, ANOVA tests were conducted. Chi-square tests were used to test for group differences in categorical variables.

Characteristic	Computer sample		Smartphone sample		χ2 or F^a^
	n	Mean (SD) or Percent	n	Mean (SD) or Percent	
Age (years)	5835	52.4 (16.6)	4778	44.9 (14.5)	595.6*
Gender					235.9*
Female	3190	55%	3310	69%	
Male	2642	45%	1465	31%	
Race					166.7*
White	4379	75%	3360	71%	
Black	471	8%	644	14%	
American Indian/Alaska Native	91	2%	124	3%	
Asian	529	9%	245	5%	
Pacific Islander	23	0%	42	1%	
Mixed	316	5%	313	7%	
Hispanic/Latino					144.2*
Yes	745	13%	1029	22%	
No	5086	87%	3748	78%	
Employment status					463.1*
Currently working	3348	57%	2795	59%	
Unemployed (laid off/looking)	303	5%	480	10%	
Retired	1337	23%	453	9%	
Disabled	194	3%	326	7%	
Other	650	11%	722	15%	
Job involving computers most of the time (if employed)					169.2*
Yes	2648	79%	1854	65%	
No	725	21%	998	35%	
Education					691.4*
High school grad or less	731	13%	1294	27%	
Some college	1137	19%	1144	24%	
Associate’s degree	638	11%	740	15%	
Bachelor’s degree	1820	31%	987	21%	
Graduate degree	1506	26%	611	13%	
Income					386.9*
< $50,000	1706	29%	2238	47%	
$50,000-$99,999	1946	33%	1366	29%	
$100,000-$149,999	1048	18%	645	14%	
≥ $150,000	1120	19%	522	11%	
Language					47.82*
English	5807	100%	4686	98%	
Spanish	28	0%	92	2%	

^a^Test of whether the computer and smartphone samples differ by the indicated characteristic.

*p < 0.05.

**Table 2:** Functional and diagnostic characteristics for the computer and smartphone samples.

	Computer			Smartphone				Computer, > = Age 65			Phone, > = Age 65			
Characteristic	Total n	Yes n	Yes (%)	Total n	Yes n	Yes (%)	χ2^a^	Total n	Yes n	Yes (%)	Total n	Yes n	Yes (%)	χ2^a^
*BADLs (binary)*														
Difficulty dressing	5277	169	3.2	4334	173	4	4.09*	1498	88	5.9	504	38	8	1.5
Difficulty eating	5277	39	0.7	4334	55	1.3	6.36*	1498	16	1.1	504	10	2.0	1.81
Difficulty walking	5276	88	1.7	4334	119	2.7	12.61*	1497	44	2.9	504	25	5.0	4.04*
Difficulty getting in/out of bed	5277	122	2.3	4334	180	4.2	25.91*	1498	52	3.5	504	21	4.2	0.34
Difficulty using toilet	5277	89	1.7	4334	135	3.1	20.7*	1498	45	3.0	504	26	5.2	4.51*
Difficulty bathing	5277	135	2.6	4334	155	3.6	8.08*	1498	63	4.2	504	32	6.3	3.37
At least 1 BADL difficulty	5276	315	6	4334	374	8.6	24.88*	1497	162	11	504	68	13.5	2.39
*IADLs (binary)*														
Difficulty with meal preparation	5277	94	1.8	4333	104	2.4	4.21*	1498	38	2.5	503	16	3.2	0.38
Difficulty making phone calls	5279	53	1	4334	68	1.6	5.67*	1498	11	1	504	7	1.4	1.15
Difficulty shopping for groceries	5278	184	3.5	4334	223	5.1	15.75*	1498	78	5.2	504	37	7.3	2.79
Difficulty taking medications	5279	52	1	4334	83	1.9	14.2*	1498	8	1	504	6	1.2	1.49
Difficulty with house/yard work	5268	483	9.2	4322	436	10.1	2.21	1492	269	18.0	500	105	21.0	1.98
Difficulty with money management	5276	73	1.4	4333	140	3.2	36.61*	1498	15	1.0	504	13	2.6	5.71*
At least 1 IADL difficulty reported	5270	632	12	4325	647	15	17.85*	1494	309	21	501	118	24	1.67
*Self-reported diagnoses (binary)*														
Diabetes	5830	896	15.4	4774	770	16.1	1.09	1624	401	24.7	531	171	32.2	11.2*
Arthritis	5830	1578	27.1	4776	1086	22.7	25.92*	1624	859	52.9	531	284	53.5	0.03
Stroke	5831	128	2.2	4776	99	2.1	0.13	1624	76	4.7	531	33	6.2	1.66
Dementia	5826	30	0.5	4768	38	0.8	2.84	1622	15	0.9	530	8	1.5	0.8
Heart condition	5831	552	9.5	4775	360	7.5	12.16*	1624	339	20.9	531	106	20.0	0.15
Hypertension	5830	2173	37.3	4775	1530	32	31.38*	1624	994	61.2	531	326	61	0
Cancer	5831	538	9.2	4774	291	6.1	35.28*	1624	356	21.9	531	116	21.8	0
Lung disease	5831	214	3.7	4776	247	5.2	13.88*	1624	114	7.0	531	52	9.8	3.95*
Emotional/psychiatric problem	5830	1122	19.2	4776	1212	25.4	57.15*	1624	184	11.3	531	96	18.1	15.53*

^a^Test of whether the computer and smartphone samples differ by the indicated characteristic.

*p < 0.05.

**Fig. 1:**
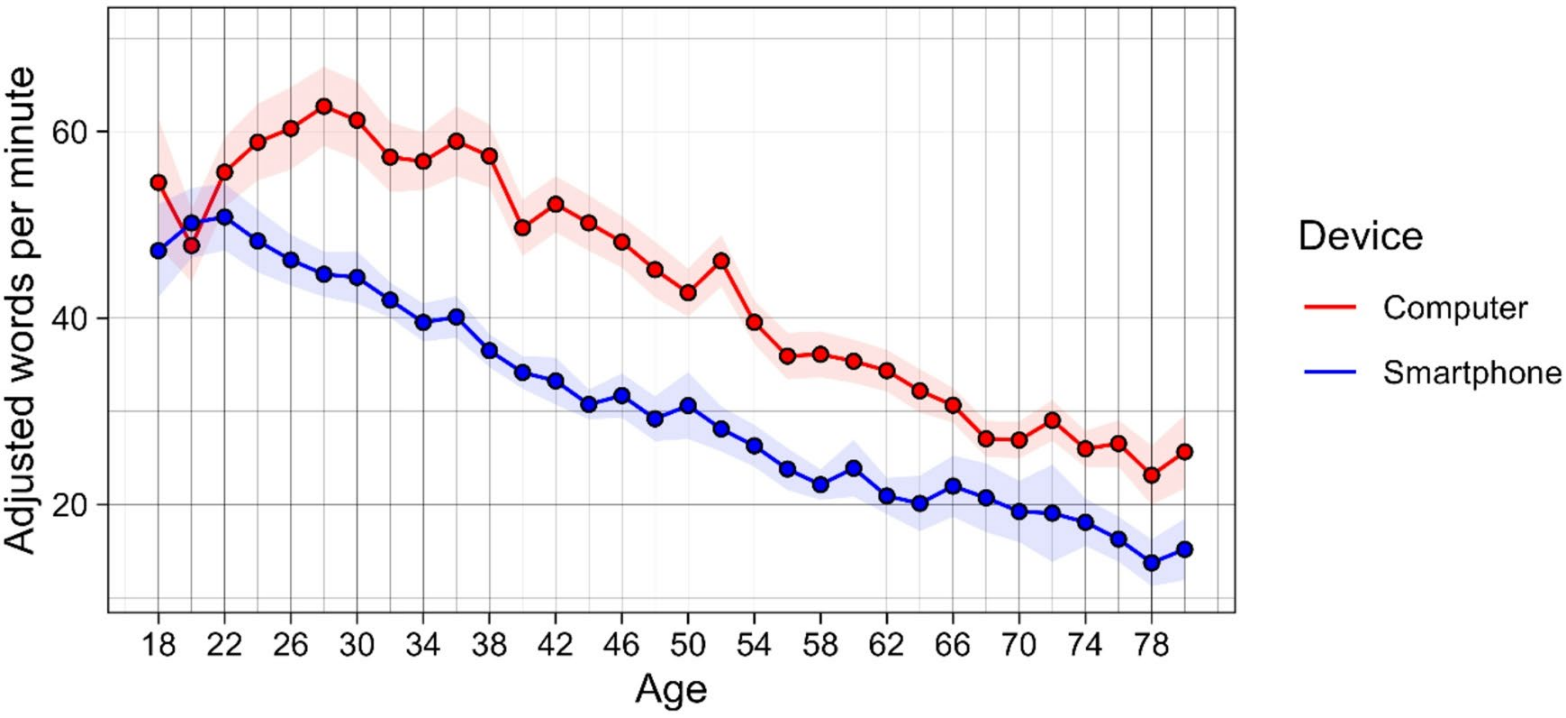
Mean typing speed for different two- year age bins (e.g. 18–19, 20–21, etc.) by device type. The bands around the trend line indicate the 95% confidence interval for the mean typing speed estimate for each age bin.

Typing speed on a computer was found to be faster than typing speed on a smartphone (*t*(10,435) = 19.3, *p* < 0.001). The difference remained when adjusting for demographic variables (β = 0.29, *p* < 0.001). For computers, the quartiles for adjusted typing speed were 25th percentile = 24.1 AWPM, median = 37.8 AWPM, and 75th percentile = 55.8 AWPM. For smartphones, they were 25th percentile = 20.0 AWPM, median = 29.4 AWPM, and 75th percentile = 42.6 AWPM. As shown in Fig. 1, AWPM was faster on computers compared to smartphones across all ages, and showed a steady decline with age (r =  − 0.52, *p* < 0.001 for computers and r =  − 0.57, *p* < 0.001 for phones). For the subsample of participants that completed the computer typing speed test twice (n = 1909), the between-person estimate for speed regressed on age was B =  − 0.021 (*p* < 0.001) while the within-person estimate was B =  − 0.015 (*p* < 0.001), but they were not statistically significantly different from one another (*p* = 0.216). For smartphones (n = 1636), the between-person estimate was B =  − 0.021 (*p* < 0.001) while the within-person estimate was B = 0.008 (*p* = 0.164), and they were statistically significantly different from one another (*p* < 0.001).

### Stability

In analyses of data from all participants who completed the typing test twice on a computer, typing speed showed an ICC of 0.79 (n = 1909) suggesting very high retest stability. For participants who completed the typing test twice on a smartphone, typing speed showed an ICC of 0.63 (n = 1636) suggesting substantial stability^28^.

### Correlations with functioning relevant measures

Correlations with smartphone typing speed were similar in magnitude and direction to correlations with computer typing speed (Table 3). For computer users, faster AWPM was associated with better cognitive functioning across a wide array of domains, less reported difficulties with BADLs and IADLs, a lower likelihood of reporting several diagnoses, lower age, better eyesight, and better self-rated computer/smartphone skills (Table 3). The magnitudes of the correlations with cognitive functioning ranged from moderate to large, with the largest correlations observed between computer AWPM and the composite perceptual speed score (r = 0.52, *p* < 0.001) and between smartphone AWPM and the composite perceptual speed score (r = 0.56, *p* < 0.001). Correlations with self-reported BADLs, IADLs, and self-reported diagnoses were generally small in magnitude. Computer typing speed had moderate correlations with self-rated computer skills (r = 0.44, *p* < 0.001) and smartphone skills (r = 0.42, *p* < 0.001).

**Table 3:** Correlations between typing speed (in adjusted words per minute, or AWPM) and other study measures by device type. All cognitive tests were scored in the direction that higher scores indicate better cognitive functioning.

	Computer AWPM					Smartphone AWPM				
	r	*p*	Adj. p	SE	95% CI	r	*p*	Adj. p	SE	95% CI
*Cognition*										
Figure ID (perceptual speed)	0.49	< 0.001	< 0.001	0.01	[0.47, 0.51]	0.48	< 0.001	< 0.001	0.01	[0.45, 0.5]
Stop and Go baseline (choice reaction time, baseline)	− 0.43	< 0.001	< 0.001	0.01	[− 0.46, − 0.41]	− 0.47	< 0.001	< 0.001	0.01	[− 0.49, − 0.44]
Stop and Go reverse (response inhibition)	− 0.42	< 0.001	< 0.001	0.01	[− 0.45, − 0.4]	− 0.44	< 0.001	< 0.001	0.01	[− 0.47, − 0.42]
Stop and Go non-switch (task switching)	− 0.48	< 0.001	< 0.001	0.01	[− 0.5, − 0.46]	− 0.52	< 0.001	< 0.001	0.01	[− 0.54, − 0.49]
Stop and Go switch (task switching)	− 0.39	< 0.001	< 0.001	0.01	[− 0.42, − 0.37]	− 0.4	< 0.001	< 0.001	0.01	[− 0.42, − 0.37]
Serial 7 (attention and working memory)	0.15	< 0.001	< 0.001	0.01	[0.12, 0.17]	0.13	< 0.001	< 0.001	0.01	[0.1, 0.16]
Number series (fluid intelligence)	0.28	< 0.001	< 0.001	0.01	[0.25, 0.3]	0.2	< 0.001	< 0.001	0.01	[0.17, 0.22]
Verbal analogies (fluid intelligence)	0.32	< 0.001	< 0.001	0.01	[0.3, 0.35]	0.24	< 0.001	< 0.001	0.01	[0.21, 0.27]
Picture vocabulary (word knowledge)	− 0.03	0.035	0.036	0.01	[− 0.05, 0]	− 0.1	< 0.001	< 0.001	0.01	[− 0.13, − 0.08]
Immediate word recall (episodic memory)	0.27	< 0.001	< 0.001	0.01	[0.24, 0.29]	0.19	< 0.001	< 0.001	0.01	[0.16, 0.22]
Delayed word recall (episodic memory)	0.27	< 0.001	< 0.001	0.01	[0.24, 0.29]	0.18	< 0.001	< 0.001	0.01	[0.15, 0.21]
Box clicking test (visual motor integration)	0.47	< 0.001	< 0.001	0.01	[0.45, 0.49]	0.37	< 0.001	< 0.001	0.01	[0.35, 0.4]
General cognitive ability composite score	0.24	< 0.001	< 0.001	0.01	[0.22, 0.26]	0.14	< 0.001	< 0.001	0.01	[0.11, 0.17]
Perceptual speed composite score	0.52	< 0.001	< 0.001	0.01	[0.5, 0.55]	0.56	< 0.001	< 0.001	0.01	[0.54, 0.59]
Memory composite score	0.28	< 0.001	< 0.001	0.01	[0.26, 0.3]	0.19	< 0.001	< 0.001	0.01	[0.17, 0.22]
*BADLs (binary)*										
Difficulty dressing	− 0.14	< 0.001	< 0.001	0.01	[− 0.17, − 0.11]	− 0.15	< 0.001	< 0.001	0.02	[− 0.18, − 0.12]
Difficulty eating	− 0.07	< 0.001	< 0.001	0.01	[− 0.1, − 0.04]	− 0.08	< 0.001	< 0.001	0.02	[− 0.11, − 0.04]
Difficulty walking	− 0.1	< 0.001	< 0.001	0.01	[− 0.13, − 0.07]	− 0.11	< 0.001	< 0.001	0.01	[− 0.14, − 0.08]
Difficulty getting in/out of bed	− 0.09	< 0.001	< 0.001	0.01	[− 0.12, − 0.06]	− 0.12	< 0.001	< 0.001	0.02	[− 0.15, − 0.09]
Difficulty using toilet	− 0.08	< 0.001	< 0.001	0.01	[− 0.11, − 0.06]	− 0.14	< 0.001	< 0.001	0.01	[− 0.17, − 0.11]
Difficulty bathing	− 0.12	< 0.001	< 0.001	0.01	[− 0.15, − 0.1]	− 0.13	< 0.001	< 0.001	0.02	[− 0.17, − 0.1]
At least 1 BADL difficulty reported	− 0.17	< 0.001	< 0.001	0.01	[− 0.2, − 0.14]	− 0.18	< 0.001	< 0.001	0.02	[− 0.21, − 0.15]
Number of BADL difficulties reported	− 0.15	< 0.001	< 0.001	0.01	[− 0.18, − 0.12]	− 0.17	< 0.001	< 0.001	0.01	[− 0.2, − 0.14]
*IADLs (binary)*										
Difficulty with meal preparation	− 0.06	< 0.001	< 0.001	0.01	[− 0.08, − 0.03]	− 0.11	< 0.001	< 0.001	0.01	[− 0.14, − 0.08]
Difficulty making phone calls	− 0.02	0.337	0.341	0.02	[− 0.05, 0.02]	0	0.961	0.961	0.01	[− 0.03, 0.03]
Difficulty shopping for groceries	− 0.1	< 0.001	< 0.001	0.01	[− 0.12, − 0.07]	− 0.14	< 0.001	< 0.001	0.02	[− 0.17, − 0.11]
Difficulty taking medications	− 0.03	0.025	0.026	0.01	[− 0.06, 0]	− 0.07	< 0.001	< 0.001	0.02	[− 0.1, − 0.03]
Difficulty with house/yard work	− 0.19	< 0.001	< 0.001	0.01	[− 0.22, − 0.16]	− 0.18	< 0.001	< 0.001	0.02	[− 0.22, − 0.15]
Difficulty with money management	− 0.04	0.004	0.004	0.01	[− 0.07, − 0.01]	− 0.05	0.002	0.002	0.02	[− 0.08, − 0.02]
At least 1 IADL difficulty reported	− 0.19	< 0.001	< 0.001	0.01	[− 0.21, − 0.16]	− 0.18	< 0.001	< 0.001	0.02	[− 0.21, − 0.15]
Number of IADL difficulties reported	− 0.16	< 0.001	< 0.001	0.01	[− 0.18, − 0.13]	− 0.17	< 0.001	< 0.001	0.02	[− 0.21, − 0.14]
Number of IADL OR BADL difficulties reported	− 0.17	< 0.001	< 0.001	0.01	[− 0.2, − 0.14]	− 0.2	< 0.001	< 0.001	0.01	[− 0.23, − 0.17]
*Self-reported diagnoses (binary)*										
Diabetes	− 0.18	< 0.001	< 0.001	0.01	[− 0.21, − 0.16]	− 0.22	< 0.001	< 0.001	0.01	[− 0.25, − 0.19]
Arthritis	− 0.28	< 0.001	< 0.001	0.01	[− 0.3, − 0.25]	− 0.25	< 0.001	< 0.001	0.01	[− 0.28, − 0.23]
Stroke	− 0.12	< 0.001	< 0.001	0.01	[− 0.14, − 0.09]	− 0.11	< 0.001	< 0.001	0.01	[− 0.14, − 0.08]
Dementia	− 0.04	0.001	0.001	0.01	[− 0.06, − 0.02]	− 0.06	0.002	0.002	0.02	[− 0.09, − 0.02]
Heart condition)	− 0.18	< 0.001	< 0.001	0.01	[− 0.2, − 0.15]	− 0.18	< 0.001	< 0.001	0.01	[− 0.21, − 0.15]
Hypertension	− 0.28	< 0.001	< 0.001	0.01	[− 0.31, − 0.26]	− 0.24	< 0.001	< 0.001	0.01	[− 0.27, − 0.22]
Cancer	− 0.14	< 0.001	< 0.001	0.01	[− 0.17, − 0.12]	− 0.15	< 0.001	< 0.001	0.01	[− 0.18, − 0.12]
Lung disease	− 0.12	< 0.001	< 0.001	0.01	[− 0.15, − 0.1]	− 0.14	< 0.001	< 0.001	0.01	[− 0.17, − 0.12]
Emotional/psychiatric problem	0.11	< 0.001	< 0.001	0.01	[0.09, 0.14]	0.06	< 0.001	< 0.001	0.01	[0.03, 0.08]
*Other*										
Age	− 0.52	< 0.001	< 0.001	0.01	[− 0.54, − 0.51]	− 0.57	< 0.001	< 0.001	0.01	[− 0.59, − 0.54]
Eyesight quality	− 0.16	< 0.001	< 0.001	0.01	[− 0.19, − 0.14]	− 0.14	< 0.001	< 0.001	0.01	[− 0.17, − 0.11]
Computer use skill	0.44	< 0.001	< 0.001	0.01	[0.42, 0.46]	0.3	< 0.001	< 0.001	0.01	[0.27, 0.32]
Smartphone use skill	0.42	< 0.001	< 0.001	0.01	[0.39, 0.44]	0.33	< 0.001	< 0.001	0.01	[0.3, 0.36]

Adj. p, Benjamini–Hochberg adjusted *p* value; BADL , basic activities of daily living; IADL , instrumental activities of daily living.

Adjusted correlations with smartphone typing speed were similar to adjusted correlations with computer typing speed. All demographic variables were associated with typing speed in bivariate models (Supplementary Text S2). We found that for both computers and smartphones, faster typing speed was associated with more years of education, higher income, and being employed (Supplementary Text S2). After adjustment for demographic variables, correlations between computer typing speed and cognitive measures ranged from r = 0.10 to r = 0.28 (Table 4). Correlations with BADLs, IADLs, and self-reported diagnoses diminished and several were no longer significant, once corrected for demographics.

**Table 4:** Correlations between typing speed (in adjusted words per minute, or AWPM) and other study measures by device type after adjustment for age, gender, race, ethnicity, working status, education, income, and preferred language.

	Computer AWPM					Smartphone AWPM				
	r	*p*	Adj. p	SE	95% CI	r	*p*	Adj. p	SE	95% CI
*Cognition*										
Figure ID (perceptual speed)	0.22	< 0.001	< 0.001	0.01	[0.2, 0.25]	0.24	< 0.001	< 0.001	0.02	[0.21, 0.27]
Stop and Go baseline (choice reaction time, baseline)	0.2	< 0.001	< 0.001	0.01	[0.17, 0.23]	0.22	< 0.001	< 0.001	0.02	[0.18, 0.25]
Stop and Go reverse (response inhibition)	0.2	< 0.001	< 0.001	0.01	[0.17, 0.23]	0.21	< 0.001	< 0.001	0.02	[0.18, 0.25]
Stop and Go non-switch (task switching)	0.23	< 0.001	< 0.001	0.02	[0.2, 0.26]	0.26	< 0.001	< 0.001	0.02	[0.23, 0.3]
Stop and Go switch (task switching)	0.18	< 0.001	< 0.001	0.01	[0.15, 0.21]	0.2	< 0.001	< 0.001	0.02	[0.17, 0.23]
Serial 7 (attention and working memory)	0.1	< 0.001	< 0.001	0.01	[0.08, 0.13]	0.09	< 0.001	< 0.001	0.01	[0.06, 0.12]
Number series (fluid intelligence)	0.17	< 0.001	< 0.001	0.01	[0.15, 0.2]	0.1	< 0.001	< 0.001	0.02	[0.07, 0.13]
Verbal analogies (fluid intelligence)	0.22	< 0.001	< 0.001	0.01	[0.2, 0.25]	0.16	< 0.001	< 0.001	0.01	[0.13, 0.19]
Picture vocabulary (word knowledge)	0.16	< 0.001	< 0.001	0.01	[0.14, 0.19]	0.08	< 0.001	< 0.001	0.02	[0.05, 0.11]
Immediate word recall (episodic memory)	0.19	< 0.001	< 0.001	0.01	[0.17, 0.22]	0.15	< 0.001	< 0.001	0.01	[0.12, 0.18]
Delayed word recall (episodic memory)	0.2	< 0.001	< 0.001	0.01	[0.17, 0.22]	0.14	< 0.001	< 0.001	0.01	[0.11, 0.17]
Box clicking test (visual motor integration)	0.28	< 0.001	< 0.001	0.01	[0.26, 0.31]	0.22	< 0.001	< 0.001	0.02	[0.19, 0.25]
General cognitive ability composite score	0.24	< 0.001	< 0.001	0.01	[0.22, 0.27]	0.15	< 0.001	< 0.001	0.02	[0.12, 0.18]
Perceptual speed composite score	0.26	< 0.001	< 0.001	0.01	[0.23, 0.29]	0.3	< 0.001	< 0.001	0.02	[0.26, 0.33]
Memory composite score	0.2	< 0.001	< 0.001	0.01	[0.18, 0.23]	0.16	< 0.001	< 0.001	0.01	[0.13, 0.19]
*BADLs (binary)*										
Difficulty dressing	− 0.05	0.001	0.002	0.01	[− 0.08, − 0.02]	− 0.05	0.002	0.003	0.02	[− 0.08, − 0.02]
Difficulty eating	− 0.04	0.007	0.011	0.01	[− 0.07, − 0.01]	− 0.03	0.067	0.078	0.02	[− 0.06, 0]
Difficulty walking	− 0.04	0.024	0.032	0.01	[− 0.06, − 0.01]	− 0.03	0.104	0.117	0.02	[− 0.06, 0.01]
Difficulty getting in/out of bed	− 0.03	0.035	0.045	0.01	[− 0.06, 0]	− 0.04	0.009	0.013	0.02	[− 0.07, − 0.01]
Difficulty using toilet	− 0.02	0.111	0.124	0.01	[− 0.05, 0.01]	− 0.06	< 0.001	< 0.001	0.02	[− 0.09, − 0.03]
Difficulty bathing	− 0.05	0.001	0.002	0.02	[− 0.08, − 0.02]	− 0.05	0.004	0.006	0.02	[− 0.08, − 0.01]
At least 1 BADL difficulty reported	− 0.06	< 0.001	< 0.001	0.01	[− 0.08, − 0.03]	− 0.05	0.001	0.002	0.02	[− 0.08, − 0.02]
Number of BADL difficulties reported	− 0.06	< 0.001	< 0.001	0.01	[− 0.09, − 0.03]	− 0.06	< 0.001	< 0.001	0.01	[− 0.09, − 0.03]
*IADLs (binary)*										
Difficulty with meal preparation	0.01	0.584	0.612	0.02	[− 0.02, 0.04]	− 0.05	0.003	0.005	0.02	[− 0.08, − 0.02]
Difficulty making phone calls	0	0.975	0.975	0.02	[− 0.03, 0.04]	0.02	0.125	0.138	0.01	[− 0.01, 0.05]
Difficulty shopping for groceries	− 0.01	0.445	0.472	0.01	[− 0.04, 0.02]	− 0.05	0.001	0.002	0.02	[− 0.08, − 0.02]
Difficulty taking medications	− 0.02	0.166	0.18	0.01	[− 0.05, 0.01]	− 0.05	0.001	0.002	0.01	[− 0.08, − 0.02]
Difficulty with house/yard work	− 0.04	0.01	0.014	0.02	[− 0.07, − 0.01]	− 0.04	0.015	0.021	0.02	[− 0.08, − 0.01]
Difficulty with money management	− 0.03	0.034	0.044	0.01	[− 0.06, 0]	− 0.04	0.01	0.014	0.01	[− 0.07, − 0.01]
At least 1 IADL difficulty reported	− 0.04	0.014	0.02	0.01	[− 0.07, − 0.01]	− 0.05	0.003	0.005	0.02	[− 0.08, − 0.02]
Number of IADL difficulties reported	− 0.04	0.022	0.03	0.01	[− 0.06, − 0.01]	− 0.06	< 0.001	< 0.001	0.02	[− 0.1, − 0.03]
Number of IADL OR BADL difficulties reported	− 0.05	< 0.001	< 0.001	0.01	[− 0.08, − 0.02]	− 0.07	< 0.001	< 0.001	0.01	[− 0.1, − 0.04]
*Self-reported diagnoses (binary)*										
Diabetes	− 0.05	< 0.001	< 0.001	0.01	[− 0.08, − 0.02]	− 0.06	< 0.001	< 0.001	0.01	[− 0.09, − 0.03]
Arthritis	− 0.03	0.027	0.035	0.01	[− 0.06, 0]	0	0.821	0.83	0.02	[− 0.04, 0.03]
Stroke	− 0.03	0.027	0.035	0.01	[− 0.06, 0]	− 0.03	0.037	0.047	0.01	[− 0.06, 0]
Dementia	− 0.01	0.658	0.681	0.01	[− 0.04, 0.02]	− 0.03	0.096	0.11	0.02	[− 0.06, 0.01]
Heart condition	− 0.03	0.049	0.058	0.01	[− 0.06, 0]	− 0.04	0.005	0.008	0.01	[− 0.07, − 0.01]
Hypertension	− 0.07	< 0.001	< 0.001	0.01	[− 0.1, − 0.04]	− 0.03	0.055	0.065	0.01	[− 0.06, 0]
Cancer	0	0.807	0.826	0.01	[− 0.02, 0.03]	− 0.01	0.363	0.39	0.01	[− 0.04, 0.02]
Lung disease	− 0.03	0.038	0.047	0.01	[− 0.06, 0]	− 0.05	0.004	0.006	0.02	[− 0.08, − 0.02]
Emotional/psychiatric problem	0.04	0.005	0.008	0.01	[0.01, 0.06]	0.03	0.049	0.058	0.01	[0, 0.06]
*Other*										
Eyesight quality	− 0.06	< 0.001	< 0.001	0.01	[− 0.08, − 0.03]	− 0.03	0.041	0.05	0.01	[− 0.06, 0]
Computer use skill	0.23	< 0.001	< 0.001	0.01	[0.2, 0.26]	0.12	< 0.001	< 0.001	0.02	[0.08, 0.15]
Smartphone use skill	0.15	< 0.001	< 0.001	0.01	[0.12, 0.18]	0.1	< 0.001	< 0.001	0.02	[0.07, 0.14]

Adj. p, Benjamini–Hochberg adjusted *p* value; BADL,  basic activities of daily living; IADL,  instrumental activities of daily living.

Within the 65 and older sample, unadjusted correlations between computer/smartphone typing speed and other measures (Table 5) were found to be attenuated compared to those observed for the whole sample (Table 3).

**Table 5:** Correlations between typing speed (in adjusted words per minute, or AWPM) and other study measures by device type, only for individuals aged 65 and older. All cognitive tests were scored in the direction that higher scores indicate better cognitive functioning.

	Computer AWPM (n = 1,718)					Smartphone AWPM (n = 570)				
	r	*p*	Adj. p	SE	95% CI	r	*p*	Adj. p	SE	95% CI
*Cognition*										
Figure ID (perceptual speed)	0.23	< 0.001	< 0.001	0.02	[0.18, 0.27]	0.32	< 0.001	< 0.001	0.04	[0.23, 0.4]
Stop and Go baseline (choice reaction time, baseline)	− 0.21	< 0.001	< 0.001	0.03	[− 0.26, − 0.15]	− 0.28	< 0.001	< 0.001	0.04	[− 0.37, − 0.19]
Stop and Go reverse (response inhibition)	− 0.2	< 0.001	< 0.001	0.03	[− 0.26, − 0.15]	− 0.26	< 0.001	< 0.001	0.04	[− 0.34, − 0.17]
Stop and Go non-switch (task switching)	− 0.22	< 0.001	< 0.001	0.03	[− 0.28, − 0.16]	− 0.34	< 0.001	< 0.001	0.04	[− 0.43, − 0.26]
Stop and Go switch (task switching)	− 0.18	< 0.001	< 0.001	0.03	[− 0.23, − 0.13]	− 0.3	< 0.001	< 0.001	0.05	[− 0.4, − 0.21]
Serial 7 (attention and working memory)	0.16	< 0.001	< 0.001	0.03	[0.11, 0.2]	0.16	< 0.001	< 0.001	0.04	[0.08, 0.25]
Number series (fluid intelligence)	0.23	< 0.001	< 0.001	0.02	[0.18, 0.28]	0.15	0.001	0.002	0.04	[0.06, 0.24]
Verbal analogies (fluid intelligence)	0.3	< 0.001	< 0.001	0.02	[0.26, 0.35]	0.22	< 0.001	< 0.001	0.04	[0.14, 0.31]
Picture vocabulary (word knowledge)	0.19	< 0.001	< 0.001	0.02	[0.14, 0.24]	0.13	0.004	0.007	0.05	[0.04, 0.23]
Immediate word recall (episodic memory)	0.25	< 0.001	< 0.001	0.02	[0.2, 0.3]	0.17	< 0.001	< 0.001	0.04	[0.08, 0.25]
Delayed word recall (episodic memory)	0.25	< 0.001	< 0.001	0.02	[0.2, 0.29]	0.17	< 0.001	< 0.001	0.05	[0.08, 0.26]
Box clicking test (visual motor integration)	0.34	< 0.001	< 0.001	0.02	[0.29, 0.38]	0.2	< 0.001	< 0.001	0.05	[0.09, 0.3]
General cognitive ability composite score	0.3	< 0.001	< 0.001	0.02	[0.26, 0.35]	0.21	< 0.001	< 0.001	0.05	[0.12, 0.3]
Perceptual speed composite score	0.25	< 0.001	< 0.001	0.03	[0.19, 0.31]	0.39	< 0.001	< 0.001	0.05	[0.3, 0.48]
Memory composite score	0.26	< 0.001	< 0.001	0.02	[0.21, 0.31]	0.18	< 0.001	< 0.001	0.04	[0.09, 0.27]
*BADLs (binary)*										
Difficulty dressing (169/5277 = 3.2% yes)	− 0.05	0.072	0.094	0.03	[− 0.1, 0]	− 0.17	< 0.001	< 0.001	0.04	[− 0.25, − 0.08]
Difficulty eating (37/5277 = 0.7% yes)	− 0.06	0.013	0.019	0.03	[− 0.11, − 0.01]	− 0.08	0.102	0.131	0.05	[− 0.17, 0.02]
Difficulty walking (90/5276 = 1.7% yes)	− 0.06	0.015	0.021	0.03	[− 0.11, − 0.01]	− 0.06	0.112	0.142	0.04	[− 0.13, 0.01]
Difficulty getting in/out of bed (121/5277 = 2.3% yes)	− 0.01	0.767	0.803	0.02	[− 0.05, 0.04]	− 0.14	0.001	0.002	0.04	[− 0.23, − 0.06]
Difficulty using toilet (90/5277 = 1.7% yes)	− 0.01	0.608	0.659	0.02	[− 0.05, 0.03]	− 0.1	0.027	0.037	0.04	[− 0.19, − 0.01]
Difficulty bathing (132/5277 = 2.5% yes)	− 0.08	0.002	0.004	0.03	[− 0.13, − 0.03]	− 0.1	0.033	0.045	0.05	[− 0.19, − 0.01]
At least 1 BADL difficulty reported (317/5276 = 6% yes)	− 0.07	0.006	0.009	0.03	[− 0.13, − 0.02]	− 0.18	< 0.001	< 0.001	0.04	[− 0.26, − 0.1]
Number of BADL difficulties reported	− 0.07	0.005	0.008	0.02	[− 0.12, − 0.02]	− 0.15	< 0.001	< 0.001	0.04	[− 0.24, − 0.07]
*IADLs (binary)*										
Difficulty with meal preparation (95/5277 = 1.8% yes)	0	0.98	0.98	0.02	[− 0.04, 0.05]	− 0.06	0.216	0.263	0.05	[− 0.15, 0.03]
Difficulty making phone calls (53/5279 = 1.0% yes)	− 0.01	0.784	0.811	0.03	[− 0.07, 0.05]	0	0.968	0.979	0.04	[− 0.08, 0.09]
Difficulty shopping for groceries (185/5278 = 3.5% yes)	− 0.06	0.023	0.032	0.03	[− 0.11, − 0.01]	− 0.11	0.006	0.009	0.04	[− 0.19, − 0.03]
Difficulty taking medications (53/5279 = 1.0% yes)	− 0.02	0.143	0.179	0.01	[− 0.05, 0.01]	− 0.08	0.185	0.228	0.06	[− 0.21, 0.04]
Difficulty with house/yard work (485/5268 = 9.2% yes)	− 0.09	0.001	0.002	0.03	[− 0.14, − 0.04]	− 0.12	0.011	0.016	0.04	[− 0.2, − 0.03]
Difficulty with money management (74/5276 = 1.4% yes)	− 0.03	0.287	0.331	0.02	[− 0.07, 0.02]	− 0.02	0.682	0.722	0.05	[− 0.11, 0.07]
At least 1 IADL difficulty reported (632/5270 = 12% yes)	− 0.1	< 0.001	< 0.001	0.03	[− 0.15, − 0.05]	− 0.12	0.007	0.011	0.04	[− 0.21, − 0.03]
Number of IADL difficulties reported	− 0.08	0.001	0.002	0.03	[− 0.13, − 0.04]	− 0.13	0.002	0.004	0.04	[− 0.22, − 0.05]
Number of IADL OR BADL difficulties reported	− 0.09	< 0.001	< 0.001	0.02	[− 0.13, − 0.04]	− 0.17	< 0.001	< 0.001	0.04	[− 0.26, − 0.09]
*Self-reported diagnoses (binary)*										
Diabetes (769/4774 = 16.1% yes)	− 0.12	< 0.001	< 0.001	0.02	[− 0.16, − 0.07]	− 0.13	0.001	0.002	0.04	[− 0.21, − 0.05]
Arthritis (1084/4776 = 22.7% yes)	− 0.03	0.258	0.306	0.03	[− 0.08, 0.02]	− 0.01	0.863	0.883	0.04	[− 0.09, 0.08]
Stroke (100/4776 = 2.1% yes)	− 0.06	0.011	0.016	0.02	[− 0.11, − 0.01]	− 0.12	0.004	0.007	0.04	[− 0.2, − 0.04]
Dementia (38/4768 = 0.8% yes)	− 0.02	0.479	0.526	0.02	[− 0.06, 0.03]	− 0.1	0.064	0.085	0.06	[− 0.22, 0.01]
Heart condition (358/4775 = 7.5% yes)	− 0.11	< 0.001	< 0.001	0.03	[− 0.16, − 0.06]	− 0.12	0.005	0.008	0.04	[− 0.2, − 0.03]
Hypertension (1528/4775 = 32.0% yes)	− 0.12	< 0.001	< 0.001	0.02	[− 0.16, − 0.07]	− 0.09	0.036	0.048	0.04	[− 0.17, − 0.01]
Cancer (291/4774 = 6.1% yes)	− 0.03	0.277	0.324	0.03	[− 0.08, 0.02]	− 0.02	0.653	0.7	0.04	[− 0.1, 0.06]
Lung disease (248/4776 = 5.2% yes)	− 0.06	0.015	0.021	0.02	[− 0.11, − 0.01]	− 0.05	0.301	0.343	0.05	[− 0.16, 0.05]
Emotional/psychiatric problem (1213/4776 = 25.4% yes)	0.03	0.258	0.306	0.03	[− 0.02, 0.08]	0.03	0.477	0.526	0.04	[− 0.05, 0.1]
*Other*										
Age	− 0.2	< 0.001	< 0.001	0.03	[− 0.25, − 0.15]	− 0.15	0.001	0.002	0.04	[− 0.23, − 0.06]
Eyesight quality	− 0.12	< 0.001	< 0.001	0.02	[− 0.17, − 0.07]	− 0.04	0.39	0.439	0.05	[− 0.13, 0.05]
Computer use skill	0.32	< 0.001	< 0.001	0.02	[0.27, 0.36]	0.22	< 0.001	< 0.001	0.05	[0.12, 0.31]
Smartphone use skill	0.26	< 0.001	< 0.001	0.03	[0.21, 0.31]	0.2	< 0.001	< 0.001	0.05	[0.11, 0.29]

Adj. p, Benjamini–Hochberg adjusted *p* value; BADL, basic activities of daily living; IADL, instrumental activities of daily living.

After Benjamin Hochberg *p*-value adjustment (Tables 3, 4, and 5), most all associations remained statistically significant.

## Discussion

Despite the brevity of the single sentence typing speed test, results demonstrated it to be a measure with sensitivity to a wide array of functioning relevant measures, supporting its use as a metric of typing skill and a digital biomarker. Even with an average gap of two years between typing tests, the test–retest stability of typing speed measurements on both computers and smartphones was high. Both computer and smartphone typing speed showed moderate to high correlations with several cognitive functioning measures, and were most highly correlated with composite perceptual speed score. Correlations with BADL/IADL status and self-reported diagnoses were generally low but in the expected direction. While both cognitive/visual and motor skills have been described as key aspects of typing performance^2,11^, in results here the associations with cognitive visual skills may have been more prominent because they were more directly measured compared to motor skills. That is, instead of direct measures of fine motor skills, our self-report measures of BADs, IADLs, and diagnoses (e.g., arthritis) served as indirect representations of fine motor capacity.

The attenuation of the correlations between typing speed and functioning relevant variables after adjustment for demographic variables may suggest that better typing skill is one form of human capital enjoyed by more privileged demographic groups that can lead to more positive functioning outcomes. That is, the effect of typing speed on diagnoses may not be spurious but a mediation pathway starting with demographics, but further research is needed to investigate this claim.

Unadjusted typing speed (i.e. AWPM) has meaning in itself as a metric of daily functioning, but researchers should consider how it is being modeled if they wish to use it to infer information about participants’ abilities. Without adjustment for demographic characteristics, typing speed may be largely representative of perceptual speed, as evidenced by its larger associations with speed as compared to other constructs such as memory and functional status. After adjustment for demographics, correlations with perceptual speed decreased greatly, likely because age is highly correlated with perceptual speed^35^. If using demographic characteristics such as age as control variables, results here suggested that typing speed may then represent some combination of general cognition and technology skill.

For the whole sample, some correlations were stronger with typing speed from the computer test as compared to the smartphone version, specifically with cognitive measures. One possible reason is that smartphones may allow the typing speed test to be completed in a wider array of environments with greater potential for distraction compared to computers (e.g., on the bus, in a waiting room for an appointment, etc.)^36^. The distraction may lead to greater noise in smartphone measured typing speed, thereby attenuating its correlations with some other measures. Consistent with the argument for a greater possibility for distraction in smartphone testing, the test–retest stability of typing speed was lower for smartphones as compared to computers. Another possible reason is that the smartphone sample was statistically significantly younger than the computer sample. Several domains of cognition decline with age^37^. Thus, the computer sample may have had a greater representation of individuals with lower cognitive test scores in the sample relative to the phone sample, leading to a greater correlation between computer based typing speed and cognitive measures as compared to the correlations observed with smartphone based typing speed.

In the 65 and older sample, correlations between computer typing speed and the cognitive composite scores were similar to one another, while the correlation between smartphone typing speed and the perceptual speed composite score was greater as compared to associations with the other composites. For the computer sample, correlations between computer typing speed and the cognitive composite scores may have been similar to one another because the association between typing speed and perceptual speed was greatly attenuated (compared to the whole sample) by only examining older adults. That is, subsetting the sample to older adults may have been similar to adjusting typing speed by age. For the 65 and older smartphone sample, the correlation between smartphone typing speed and perceptual speed may have been less attenuated (and larger than correlations with the other composites) because, even with the “adjustment” for age, perceptual speed may remain a primary driver of smartphone typing speed in older adults. That is, as people become more skilled in typing on a computer, muscle memory and tactile feedback play a greater role in pressing the correct keys. However, with smartphone typing, older adults may continue to rely on perceptual speed to find letters on the digital keyboard before pressing them.

Study limitations should be noted. Participants chose which device to use for the typing test, which led to differences in the computer and smartphone typing samples (Table 1). Future studies are needed to examine the test–retest reliability (i.e., stability) of the typing speed test when completed twice with a minimal time lag (e.g., within the same day). We examined cohort relative to aging effects on typing speed, but an average of only two years between typing measures may be insufficient to fully capture aging effects. Since participants were recruited into an online panel study, there is a possibility that they may be more technologically savvy compared to the general population. While the UAS sample intends to be nationally representative of characteristics such as race and state of residence, older adults with very low functional capabilities may have been underrepresented as special efforts were not made to ensure that such individuals were recruited and could complete the online surveys. In the computer group, of adults aged 65 and older, 11% reported at least 1 BADL difficulty and 21% reported at least 1 IADL difficulty.

## Conclusion

A one sentence typing speed test was found to yield a measurement of typing speed on both computers and smartphones with high stability across two years. Furthermore, study results supported the functional relevance of typing speed. With advancements in technology typing has become increasingly integral to everyday functioning. Thus, adding typing performance to the repertoire of typical functioning measures (e.g., gait speed, BADL status, etc.) may enrich understanding of everyday functioning difficulties.

## Supplementary Information

Below is the link to the electronic supplementary material.

Supplementary Material 1

## Data Availability

Quantitative data sets are available from the Understanding America Study website (http://uasdata.usc.edu). Consistent with open science practices, any individual affiliated with a research institution is able to access these data for purpose of replication or research after free registration, and provision of a signed data use agreement.

## Code availability

The software code that supports the findings of this study is available from the corresponding author, R.H., upon reasonable request.

## References

[1] Muñoz-Neira, C. et al. The technology – activities of daily living questionnaire: A version with a technology-related subscale. *Dement. Geriatr. Cogn. Disord.* **33**, 361–371 (2012). 22797087 10.1159/000338606 PMC4722866

[2] Pinet, S., Zielinski, C., Alario, F.-X. & Longcamp, M. Typing expertise in a large student population. *Cogn. Res.: Princ. Implic.* **7**, 77 (2022). 35930064 10.1186/s41235-022-00424-3 PMC9356123

[3] Liu, Y. et al. A novel cloud-based framework for the elderly healthcare services using digital twin. *IEEE Access* **7**, 49088–49101 (2019).

[4] Koch, J., Frommeyer, B. & Schewe, G. Online shopping motives during the COVID-19 pandemic—Lessons from the crisis. *Sustainability* **12**, 10247 (2020).

[5] Williams, J. R. The use of online social networking sites to nurture and cultivate bonding social capital: A systematic review of the literature from 1997 to 2018. *New Media Soc.* **21**, 2710–2729 (2019).

[6] Kim, H., Park, I., joo Lee, H. & Lee, O. The reliability and validity of gait speed with different walking pace and distances against general health, physical function, and chronic disease in aged adults. *J. Exerc. Nutr. Biochem.* **20**, 46–50 (2016). 10.20463/jenb.2016.09.20.3.7 PMC5067420 27757387

[7] Group, F.-N. B. W. FDA-NIH Biomarker Working Group BEST (biomarkers, EndpointS, and other tools) resource. *Food and Drug Administration (US), Silver Spring, MD (2016) Co-published by National Institutes of Health (US): Bethesda (MD)*[https://www.ncbi.nlm.nih.gov/books/NBK326791/*, consulted in February 2023*] (2016). 27010052

[8] Dagum, P. Digital biomarkers of cognitive function. *npj Digit. Med.* **1**, 1–3 (2018). 31304295 10.1038/s41746-018-0018-4 PMC6550173

[9] Jacobson, N. C., Summers, B. & Wilhelm, S. Digital biomarkers of social anxiety severity: Digital phenotyping using passive smartphone sensors. *J. Med. Internet Res.* **22**, e16875 (2020). 32348284 10.2196/16875 PMC7293055

[10] Seelye, A. et al. Computer mouse movement patterns: A potential marker of mild cognitive impairment. *Alzheimer’s Dement. Diagn. Assess. Dis. Monit.* **1**, 472–480 (2015). 10.1016/j.dadm.2015.09.006 PMC4748737 26878035

[11] Salthouse, T. A. Perceptual, cognitive, and motoric aspects of transcription typing. *Psychol. Bull.* **99**, 303 (1986). 3714922

[12] Mather, N. & Jaffe, L. E. *Woodcock-Johnson IV: Reports, Recommendations, and Strategies*. (John Wiley & Sons, 2016).

[13] Alfalahi, H. et al. Diagnostic accuracy of keystroke dynamics as digital biomarkers for fine motor decline in neuropsychiatric disorders: A systematic review and meta-analysis. *Sci. Rep.* **12**, 7690 (2022). 35546606 10.1038/s41598-022-11865-7 PMC9095860

[14] Summers, J., Larkin, D. & Dewey, D. Activities of daily living in children with developmental coordination disorder: Dressing, personal hygiene, and eating skills. *Hum. Mov. Sci.* **27**, 215–229 (2008). 18348898 10.1016/j.humov.2008.02.002

[15] Matarazzo, M. et al. Remote monitoring of treatment response in Parkinson’s disease: The habit of typing on a computer. *Mov. Disord.* **34**, 1488–1495 (2019). 31211469 10.1002/mds.27772

[16] Muela, H. C. S. et al. Hypertension severity is associated with impaired cognitive performance. *J. Am. Heart Assoc.* **6**, e004579 (2017). 28077386 10.1161/JAHA.116.004579 PMC5523638

[17] Yun, H.-S., Kim, E., Suh, S.-R., Kim, M.-H. & Kim, H. Diabetes reduces the cognitive function with the decrease of the visual perception and visual motor integration in male older adults. *J. Exerc. Rehabil.* **9**, 470–476 (2013). 24282807 10.12965/jer.130059 PMC3836550

[18] Ko, P.-H., Hwang, Y.-H. & Liang, H.-W. Influence of smartphone use styles on typing performance and biomechanical exposure. *Ergonomics* **59**, 821–828 (2016). 26328936 10.1080/00140139.2015.1088075

[19] Turner, C. J., Chaparro, B. S., Sogaard, I. M. & He, J. The effects of keyboard layout and size on smartphone typing performance. *Proc. Hum. Factors Ergon. Soc. Annu. Meet.* **64**, 985–989 (2020).

[20] Alattar, L., Messel, M. & Rogofsky, D. An introduction to the understanding America study internet panel. *Soc. Sec. Bull.* **78**, 13–28 (2018).

[21] Kapteyn, A., Angrisani, M., Darling, J. & Gutsche, T. The Understanding America Study (UAS). *BMJ Open* **14**, e088183 (2024). 39448221 10.1136/bmjopen-2024-088183 PMC11499792

[22] Health and Retirement Study. HRS 2018 - SECTION D: COGNITION. https://hrs.isr.umich.edu/sites/default/files/meta/2018/core/qnaire/online/04hr18D.pdf (2018).

[23] Berg, S. Psychological functioning in 70- and 75-year-old people: A study in an industrialized city. *Acta Psychiatr. Scand.* **62**, 5–47 (1980). 6935934

[24] Lachman, M. E., Agrigoroaei, S., Tun, P. A. & Weaver, S. L. Monitoring cognitive functioning: psychometric properties of the brief test of adult cognition by telephone. *Assessment* **21**, 404–417 (2014). 24322011 10.1177/1073191113508807 PMC4050038

[25] Welsh, K. A., Breitner, J. C. S. & Magruder-Habib, K. M. Detection of dementia in the elderly using telephone screening of cognitive status. *Cogn. Behav. Neurol.* **6**, 103 (1993).

[26] Hernandez, R. et al. Visual-motor integration (VMI) is also relevant for computer, smartphone, and tablet use by adults: Introducing the brief box clicking test. *Am. J. Occup. Ther.* **78**, 7805205010 (2024). 39054682 10.5014/ajot.2024.050680 PMC12309844

[27] Curran, P. J. & Bauer, D. J. The disaggregation of within-person and between-person effects in longitudinal models of change. *Annu. Rev. Psychol.* **62**, 583–619 (2011). 19575624 10.1146/annurev.psych.093008.100356 PMC3059070

[28] Bushnell, C. D., Johnston, D. C. C. & Goldstein, L. B. Retrospective assessment of initial stroke severity. *Stroke* **32**, 656–660 (2001). 11239183 10.1161/01.str.32.3.656

[29] Cohen, J. *Statistical Power Analysis for the Behavioral Sciences*. (Routledge, New York, 1988). 10.4324/9780203771587.

[30] Jafari, M. & Ansari-Pour, N. Why, when and how to adjust your P values?. *Cell J.* **20**, 604–607 (2019). 30124010 10.22074/cellj.2019.5992 PMC6099145

[31] Clegg, A., Young, J., Iliffe, S., Rikkert, M. O. & Rockwood, K. Frailty in elderly people. *Lancet* **381**, 752–762 (2013). 23395245 10.1016/S0140-6736(12)62167-9 PMC4098658

[32] Muthén, L. K. & Muthén, B. O. *Mplus user’s guide* 8th edn (Muthén & Muthén, 1998).

[33] R Core Team. R: A language and environment for statistical computing. R Foundation for Statistical Computing. https://www.r-project.org/ (2020).

[34] Hallquist, M. N. & Wiley, J. F. MplusAutomation: An R package for facilitating large-scale latent variable analyses in Mplus. *Struct. Equ. Model.* **25**, 621–638 (2018). 10.1080/10705511.2017.1402334 PMC6075832 30083048

[35] Salthouse, T. A. The processing-speed theory of adult age differences in cognition. *Psychol. Rev.* **103**, 403–428 (1996). 8759042 10.1037/0033-295x.103.3.403

[36] Akpinar, E., Yeşilada, Y. & Karagöz, P. Effect of context on smartphone users’ typing performance in the wild. *ACM Trans. Comput.-Hum. Interact.* **30**, 1–44 (2023).

[37] Deary, I. J. et al. Age-associated cognitive decline. *Br. Med. Bull.* **92**, 135–152 (2009). 19776035 10.1093/bmb/ldp033

