# Supplementary material for: Functional and cognitive correlates of typing speed in a large U.S. panel study
Source: Sci Rep. 2026 Jan 21;16:5900. doi: 10.1038/s41598-026-36500-7 (PMC12894681; doi:10.1038/s41598-026-36500-7)
Supplement: Supplementary file 1 — Supplementary Material 1 [file 41598_2026_36500_MOESM1_ESM.docx]

**Supplementary Material**

**Supplementary Text S1.** More detailed descriptions of study measures.

Typing Speed Test

- The Spanish pangram “Cada vez que trabajo, Felix me paga un whisky” utilized for the typing test translates to “Every time I work, Felix pays me a whiskey.”
- Typing speed was computed as words per minute with the following formula: (number of characters typed inclusive of spaces/5)/(minutes between first and last key press) ^1^.
- Accuracy of the typed text was computed with the formula ((number of characters in the target sentence)-(Levenshtein distance between target and typed sentence))/(number of characters in target sentence).^1^ For English speakers, the target “quick brown fox” sentence had 43 characters. Levenshtein distance is the minimum number of insertions, deletions, and/or substitutions needed to transform the typed sentence to the targeted one.^2^ If the typed and target sentences were identical to one another, then the Levenshtein distance would be 0 and accuracy 100%. Incorrect letter casing (upper vs. lower) was counted as an error. Typing speed for observations with less than 50% accuracy were set to missing for being over erroneous (2.5% of observations).^1^
- Use of copy paste, predictive text, and/or autocorrect were accounted for by setting observations to missing if they had times between keystrokes that were too short, suggesting use of these functions (1% of observations).
- Adjusted typing speed, or adjusted words per minute (AWPM), was computed as the product between typing speed and accuracy.^1^ This metric was used as the primary measure of typing speed because it considered both speed and accuracy of keyboarding. In analyses it was log-transformed to correct for positive skew in the distribution. Note that typing speed and adjusted typing speed were found to be essentially identical with a correlation of r=.998, likely because a vast majority of participants typed the single target sentence accurately.

Cognitive Tests

- Serial 7s: Assesses **sustained attention** and **working memory**.^3^ Participants are asked to sequentially subtract seven from a given starting number (e.g., 100) five times. One point is awarded for one correct subtraction, two points for two or three correct subtractions, and three points for four of five correct subtractions.
- Immediate/Delayed Word Recall: Tests **semantic** **episodic memory** with immediate and delayed word recall tasks.^4^ Participants are presented with a list of 10 words and asked to recall them immediately and after a delay of several minutes. Scoring includes the total number of correctly recalled words for each phase, with additional indicators for task non-completion or refusals. Misspellings that plausibly represented the correct word are counted as correct.
- Brief Box Clicking Test: Assesses **processing speed** and **visual motor integration**.^5^ Participants are presented with four stationary boxes, two on a top row and two immediately below. They are then asked to click (or tap) each box as quickly as they can. Note that this score is only available in HRS *waves 4 and 5*.
- Number Series: Assesses **fluid intelligence** by presenting participants with a series of numbers and asking them to identify the missing number based on pattern recognition.^6^ IRT-based normed scores were derived using a two-parameter logistic IRT model. Two parallel forms are administered in alternating order to reduce practice effects.
- Verbal Analogies: Assesses **fluid intelligence** by prompting participants to identify relationships between word pairs.^6^ IRT-based normed scores for two alternating parallel forms were derived using a two-parameter logistic IRT model.
- Picture Vocabulary: Tests **word knowledge** by presenting participants with a series of pictures and asking them to type the names of each one.^6^ IRT-based normed scores for two alternating parallel forms were derived using a two-parameter logistic IRT model.
- Stop and Go Switch Test: Participants respond to stimuli by selecting the word “stop” or “go” according to alternating reaction rules (e.g., respond “stop” when see the word “red”).^7–9^ It is divided into four trials with different reaction rules: baseline, reverse, non-switch, and switch. The trials measure **choice reaction time**, **response inhibition**, **tasking switching**, and **task switching** (again), respectively. The median response time in each type of trial is used as a person’s score, with lower times indicating greater ability. Response inhibition and task switching are relevant to **executive functioning**.
- Figure Identification Test: Assesses **perceptual speed** by presenting participants with one figure on top of 5 horizontally aligned similar figures.^7^ They are then prompted to select one figure from the bottom 5 that exactly matches the one top figure as quickly as possible while being accurate. The more figures that are correctly selected, the greater the perceptual speed score. The test uses two alternating parallel forms to reduce practice effects.

Exploratory Factor Analysis Cognitive Test Loadings

- Additionally, composite scores for general cognition, speed, and memory were computed. An exploratory factor analysis was conducted using all the cognitive measures, and model fit was evaluated with traditional cutoffs of Root Mean Square Error of Approximation (RMSEA) <0.05, comparative fit index (CFI) and Tucker–Lewis Index (TLI) >0.95, and standardized root mean square residual (SRMR) < 0.08^10^. A 3-factor model was found to have good fit (RMSEA=0.048, CFI=0.98, TLI=0.97, and SRMR=0.018) while having a theoretically supported factor structure. Supplementary Table S1 shows the factor loadings.

**Supplementary Table S1.** Factor loadings for the 3-factor model are shown below. We operationalized a test as loading strongly on a factor if it had a factor loading of 0.5 or greater.

| **Cognitive Measure** | **Factor 1 (general cognition)** | **Factor 2 (speed)** | **Factor 3 (memory)** |
| --- | --- | --- | --- |
| Serial 7 | 0.42* | -0.01 | 0.06* |
| Number Series | 0.71* | 0.04* | -0.02 |
| Verbal Analogies | 0.87* | 0 | -0.09* |
| Picture Vocabulary | 0.71* | -0.31* | 0.01* |
| Figure Identification | 0.1* | 0.6* | 0.02 |
| Stop and Go baseline | -0.02 | 0.8* | 0.01 |
| Stop and Go reverse | 0 | 0.83* | -0.01 |
| Stop and Go non-switch | -0.01 | 0.94* | -0.03* |
| Stop and Go switch | 0.1* | 0.69* | 0.01 |
| Immediate Recall | 0.02 | 0.01 | 0.84* |
| Delayed Recall | -0.01 | 0 | 0.95* |
| Box Clicking | 0.02 | 0.39* | 0.02 |

- The general intelligence factor was made up of tests relevant to fluid intelligence (Number Series, Verbal Analogies) and crystallized intelligence (Picture Vocabulary), both of which are aspects of general intelligence^11^.
- The perceptual speed factor was made up of scores on the perceptual speed test (Figure Identification) and all four scores from the Stop and Go task. While the Stop and Go task does not focus on assessing processing speed, all its measures are based on task completion time, meaning that a perceptual speed element is inherent in all its scores.
- Finally, the memory factor was made up of scores on the immediate and delayed word recall tasks.
- Note that the distinction between general intelligence, speed, and memory is supported by prior research^12,13^.
- For each of the factors, a composite score was computed by first standardizing all the cognitive measures and then computing the average of the standardized scores within each factor^14^. Prior to standardizing, all the cognitive scores were recoded so that higher scores would indicate better cognitive performance.

**References**

1. Pinet S, Zielinski C, Alario FX, Longcamp M. Typing expertise in a large student population. Cognitive Research: Principles and Implications. 2022 Aug 5;7(1):77.

2. Haldar R, Mukhopadhyay D. Levenshtein Distance Technique in Dictionary Lookup Methods: An Improved Approach [Internet]. arXiv; 2011 [cited 2024 Sep 17]. Available from: http://arxiv.org/abs/1101.1232

3. Bristow T, Jih CS, Slabich A, Gunn J. Standardization and adult norms for the sequential subtracting tasks of serial 3’s and 7’s. Applied Neuropsychology: Adult. 2016 Sep 2;23(5):372–8.

4. Runge SK, Craig BM, Jim HS. Word Recall: Cognitive Performance Within Internet Surveys. JMIR Mental Health. 2015 Jun 2;2(2):e20.

5. Hernandez R, Gatz M, Schneider S, Finkel D, Darling JE, Orriens B, et al. Visual–Motor Integration (VMI) Is Also Relevant for Computer, Smartphone, and Tablet Use by Adults: Introducing the Brief Box Clicking Test. The American Journal of Occupational Therapy. 2024 Jul 26;78(5):7805205010.

6. Mather N, Jaffe LE. Woodcock-Johnson IV: Reports, Recommendations, and Strategies. John Wiley & Sons; 2016. 617 p.

7. Liu Y, Schneider S, Orriens B, Meijer E, Darling JE, Gutsche T, et al. Self-administered Web-Based Tests of Executive Functioning and Perceptual Speed: Measurement Development Study With a Large Probability-Based Survey Panel. Journal of Medical Internet Research. 2022 May 9;24(5):e34347.

8. Lachman ME, Agrigoroaei S, Tun PA, Weaver SL. Monitoring cognitive functioning: psychometric properties of the brief test of adult cognition by telephone. Assessment. 2014 Aug 1;21(4):404–17.

9. Tun PA, Lachman ME. Age differences in reaction time and attention in a national telephone sample of adults: Education, sex, and task complexity matter. Developmental Psychology. 2008;44(5):1421–9.

10. Hu L tze, Bentler PM. Cutoff criteria for fit indexes in covariance structure analysis: Conventional criteria versus new alternatives. Structural equation modeling: a multidisciplinary journal. 1999;6(1):1–55.

11. Cattell RB. Fluid and Crystallized Intelligence. In: Studies in individual differences: The search for intelligence. East Norwalk, CT, US: Appleton-Century-Crofts; 1961. p. 738–46.

12. Horn JL. Organization of abilities and the development of intelligence. Psychological review. 1968;75(3):242.

13. Flanagan DP, Dixon SG. The Cattell-Horn-Carroll Theory of Cognitive Abilities. In: Encyclopedia of Special Education [Internet]. John Wiley & Sons, Ltd; 2014 [cited 2025 Aug 21]. Available from: https://onlinelibrary.wiley.com/doi/abs/10.1002/9781118660584.ese0431

14. Widaman KF, Revelle W. Thinking thrice about sum scores, and then some more about measurement and analysis. Behav Res Methods. 2023;55(2):788–806.

**Supplementary Table S2.** Sample characteristics for computer (n=1,909) and smartphone (n=1,636) subsamples that completed the typing speed test twice on the same device, allowing for examination of test re-test stability. To test if the samples differed by continuous variables like age, ANOVA tests were conducted. Chi-square tests were used to test for group differences in categorical variables.

|  | **Computer sample** | | **Smartphone sample** | |  |
| --- | --- | --- | --- | --- | --- |
| **Characteristic** | **n** | **Mean (SD) or Percent** | **n** | **Mean (SD) or Percent** | **χ2 or F^a^** |
| Age (years) | 1,909 | 55.2 (16.1) | 1,636 | 45.5 (13.8) | 363.1* |
| Gender |  |  |  |  | 118.2* |
| Female | 993 | 52% | 1,146 | 70% |  |
| Male | 914 | 48% | 490 | 30% |  |
| Race |  |  |  |  | 45.2* |
| White | 1,506 | 79% | 1,210 | 75% |  |
| Black | 115 | 6% | 166 | 10% |  |
| American Indian/Alaska Native | 36 | 2% | 48 | 3% |  |
| Asian | 146 | 8% | 73 | 5% |  |
| Pacific Islander | 11 | 1% | 12 | 1% |  |
| Mixed | 90 | 5% | 105 | 7% |  |
| Hispanic/Latino |  |  |  |  | 89.9* |
| Yes | 223 | 12% | 390 | 24% |  |
| No | 1,684 | 88% | 1,246 | 76% |  |
| Employment status |  |  |  |  | 239.8* |
| Currently working | 1,005 | 53% | 938 | 57% |  |
| Unemployed (laid off/looking) | 94 | 5% | 171 | 10% |  |
| Retired | 517 | 27% | 143 | 9% |  |
| Disabled | 60 | 3% | 122 | 7% |  |
| Other | 232 | 12% | 261 | 16% |  |
| Job involving computers most of the time (if employed) |  |  |  |  | 52.4* |
| Yes | 846 | 77% | 675 | 64% |  |
| No | 256 | 23% | 383 | 36% |  |
| Education |  |  |  |  | 231.3* |
| High school grad or less | 252 | 13% | 452 | 28% |  |
| Some college | 383 | 20% | 410 | 25% |  |
| Associate’s degree | 222 | 12% | 264 | 16% |  |
| Bachelor’s degree | 584 | 31% | 299 | 18% |  |
| Graduate degree | 467 | 24% | 210 | 13% |  |
| Income |  |  |  |  | 157.8* |
| <$50,000 | 581 | 31% | 832 | 51% |  |
| $50,000-$99,999 | 668 | 35% | 445 | 27% |  |
| $100,000-$149,999 | 344 | 18% | 199 | 12% |  |
| ≥$150,000 | 310 | 16% | 158 | 10% |  |
| Language |  |  |  |  | 15.3* |
| English | 1,901 | 100% | 1,606 | 98% |  |
| Spanish | 8 | 0% | 30 | 2% |  |

^a^Test of whether the computer and smartphone samples differ by the indicated characteristic.

*p<0.001

**Supplementary Text S2.** Demographic differences in computer and smartphone typing speed

In exploratory analyses we examined the nature of demographic differences in typing speed by regressing typing speed on each demographic variable individually. Computer typing speed was on average slower for males compared to females (standardized β =-0.10, p<0.001). It was also significantly different by race (p<0.001) such that Asians were faster than Whites (standardized β =0.25, p<0.001) but the typing speed of Whites was not significantly different from other racial groups. Faster computer typing speed was associated with being employed (standardized β =0.38, p<0.001), having more years of education (standardized β =0.28, p<0.001), higher income (standardized β =0.25, p<0.001), and requesting surveys in English rather than Spanish (standardized β =0.08, p<0.001). There was not a significant association between typing speed and identifying as Hispanic or not.

In terms of smartphones, phone typing speed was on average slower for males compared to females (standardized β =-0.07, p<0.001). It was also significantly different by race (p<0.001) such that Asians and those that identified as mixed race were faster than Whites (standardized β =0.09, p<0.001, and standardized β =0.04, p=0.028, respectively), but those that identified as Native American were slower (standardized β =-0.03, p=0.046). Faster computer typing speed was associated with identifying as Hispanic or Latino (standardized β =0.07, p<0.001), being employed (standardized β =0.25, p<0.001), having more years of education (standardized β =0.17, p<0.001), higher income (standardized β =0.19, p<0.001), and requesting surveys in English rather than Spanish (standardized β =0.06, p<0.001).
